# Social Media Marketing of Non-Evidence-Based Women's Health Interventions: Protocol for a Content Analysis Using Participatory Research Methods

**DOI:** 10.2196/76750

**Published:** 2025-10-14

**Authors:** Brooke Nickel, Tessa Copp, Emma Gram, Jolyn Hersch, Claire Hudson, Kathleen McFadden, Kristen Pickles, Jenna Smith, Melody Taba, Alice Graham, Becky Freeman, Barbara Mintzes, Jenny Doust, Deborah Cohen, Kirsten McCaffery

**Affiliations:** 1 Sydney Health Literacy Lab, Sydney School of Public Health, Faculty of Medicine and Health, The University of Sydney Sydney Australia; 2 Wiser Healthcare, Sydney School of Public Health, Faculty of Medicine and Health, The University of Sydney Sydney Australia; 3 Section of General Practice, Department of Public Health, University of Copenhagen Copenhagen Denmark; 4 Prevention Research Collaboration, Sydney School of Public Health, Faculty of Medicine and Health, The University of Sydney Sydney Australia; 5 Charles Perkins Centre and School of Pharmacy, Faculty of Medicine and Health, The University of Sydney Sydney Australia; 6 Australian Women and Girls’ Health Research (AWaGHR) Centre, School of Public Health, The University of Queensland Brisbane Australia; 7 London School of Economics and Political Science London United Kingdom

**Keywords:** social media, women’s health, evidence-based medicine, overdiagnosis, content analysis, participatory research methods

## Abstract

**Background:**

The promotion of non-evidence-based health interventions to women on social media is a growing problem.

**Objective:**

This study aims to explore the use of social media to disseminate and promote health interventions that lack robust evidence and are of current interest and popularity.

**Methods:**

A content analysis of posts on TikTok, Instagram, and Facebook about 5 health interventions targeted at women will be conducted using participatory research methods with consumers. English-language posts that discuss boric acid suppositories, fertility testing, perimenopause and menopause testing, supplements and hormone treatments for menopause, and menopause hormone therapy for disease prevention will be included. Using keyword searches related to each health intervention, consumers will screen the top posts until 100 eligible posts on 2 different social media platforms are identified (1000 posts total across the 5 health interventions). Data from the post’s caption, on-screen text, and audio and/or video will be included in the analysis. The analysis of these posts will take both a deductive approach using a prespecified framework and an inductive approach, generating key themes from the post content.

**Results:**

Data on TikTok, Instagram, and Facebook have been searched and screened. Development of the coding framework and analysis is now underway. The findings will be disseminated via publications in peer-reviewed international medical journals and presentations at national and international conferences in 2025 and 2026.

**Conclusions:**

This novel study will provide important insights into how information on various women’s health interventions and products, which currently lack robust evidence of benefit, are being disseminated and promoted on social media to women. Understanding this is essential for developing strategies to mitigate potential harm and plan solutions, thus protecting women from the low-value interventions marketed to them, becoming patients unnecessarily, and taking finite resources away from the health care system.

**International Registered Report Identifier (IRRID):**

DERR1-10.2196/76750

## Introduction

### Background

It is now widely acknowledged that the public and patients alike are turning to social media for health information [1]. Conveying health information on social media has both positive and negative impacts [2,3]. Social media, when used as a public communication channel by reliable sources such as health agencies, nongovernmental organizations, health professionals, and communities with chronic health conditions, typically provides greater access to important health information [4]. However, recent studies found stark differences in the accuracy of women’s health information across social media [5,6], and the accuracy of information can depend on the creator and their credentials [7]. While different platforms claim to regulate health information, current international regulations may not be geared to respond to the challenges posed by claims and marketing on social media [8,9]. Influencers and companies share health information with their audiences, even though they may not necessarily have relevant knowledge or qualifications to do so, and deliberate agendas for both mis- and disinformation exist. This increases the risk that the information they share can be misleading, incomplete, or inaccurate. Many individuals or companies also have financial conflicts of interest, which can further complicate the quality and neutrality of information [10]. In 2024, influencer marketing drove social media to become the world’s leading advertising channel, including the sale of health and wellness products, reaching almost US $250 billion in sales [11].

One particular area of concern for the dissemination of health information on social media is women’s health [12]. Women’s health has historically been underfunded and underresearched, meaning that large knowledge gaps still exist [13]. In lieu of evidence, influencers, alternative medicine practitioners, and companies are now attempting to fill this space, and social media provides a platform that facilitates the rapid and broad spread of information. Some influencers on social media, including highly influential celebrities with substantial numbers of followers, even have their own women’s health brands or businesses, or have vested interests in the interventions. This makes the landscape for sharing health information on social media even more complex because influencers receive significant financial incentives to promote the brand by strongly affirming any underlying health claims [14]. Recent examples include actress Gwyneth Paltrow with her Goop Lab products [15], media personality Kourtney Kardashian’s lemme vagina probiotics [16], and the ZOE app for menopausal women [17], which claims to be based on “world-leading science” and has been highly promoted by British television presenter Davina McCall. All of these examples, despite having no robust evidence of health benefits, are successful and seemingly popular among female consumers. However, there is still little evidence on how women’s health interventions, such as these, are being marketed to women on social media.

### This Study

While previous studies have explored women’s health information on social media [5,6], to the best of our knowledge, none have comprehensively analyzed the content and frequency of posts about non–evidence-based health interventions promoted to women and are of interest to women of different ages or life stages. Our team recently analyzed how commercial interests co-opt narratives in women’s health to promote non-evidence-based interventions [18]. However, our earlier article did not address the way social media—via influencers, celebrities, and influential companies may endorse or promote non–evidence-based women’s health interventions, making it even more difficult for women to know what information is valid and to avoid low-value care [19].

The overarching aim of this study is to explore how social media is used to disseminate and promote health information and sell health products to women, with a specific focus on 5 health interventions that lack robust evidence and are of current interest and popularity. Specifically, this study will assess how balanced the posts are in the discussion of the benefits, harms, and caveats of the intervention; the use of evidence, anecdotes, or appeal strategies in the posts; and whether the financial interests of account holders exist.

## Methods

### Study Design

A content analysis [20] of social media posts on 5 women’s health interventions will be conducted using participatory research methods with consumers. This approach strengthens the research process and improves the effectiveness and relevance of study outputs to women. Using social media platforms (TikTok, Instagram, and Facebook), we will analyze what and how information on these specific interventions is being disseminated and marketed to women. Shorter-content platforms, including TikTok, Instagram, and Facebook, are used because this type of short-form content continues to grow in popularity across all age groups [21]. The study methods follow those of a recent study using content analysis to analyze posts about health tests on social media [22,23], adapted and tailored to address the research aims and questions as outlined earlier, in partnership with consumers. Consumers are women from a diverse consumer panel (Co-SHeLL) [24] based in Australia. Consumers provide a broad range of perspectives to the study, reflecting their age and social media experience. Researchers approached the panel to assess female panel members’ interest in being involved as part of the study team, including contributions to the study design, data collection, analysis, and interpretation of the study results. Consumers were compensated AUD $50 (US$32) per hour for their time and will be offered authorship for any resulting empirical publications.

### Eligibility Criteria

English-language posts about the selected interventions were included from accounts on TikTok, Instagram, and Facebook. Each intervention included 2 platforms based on user statistics for the age group where the intervention is typically discussed and marketed, as well as the project’s consumer use preferences. The selected interventions met the following criteria: (1) evidence-based concerns that these interventions reflect low-value care (ie, the intervention does not lead to improved health outcomes for generally healthy women and may lead to harm or waste, or reduce the uptake of high-value care) and (2) currently being promoted to generally healthy women on social media. Text-only posts, images, infographics, and videos, including reels, and audio-only posts that explicitly discussed the health intervention in question were all included.

In consultation with consumers of various age ranges identifying what health topics they regularly see on their social media feeds and querying whether certain interventions with no evidence of benefit appear on their feeds, the 5 interventions included: boric acid suppositories to maintain vaginal health, fertility testing, perimenopause and menopause testing, supplements and hormone treatments for menopause, and menopause hormone therapy for disease prevention.

### Included Women’s Health Interventions

#### Boric-Acid Suppositories

Boric acid suppositories are marketed on social media as a vaginal health product designed to cleanse and refresh after menstruation, balance pH levels, improve vaginal taste and odor, and prevent and treat yeast infections or bacterial vaginosis [25]. Although boric acid has antibacterial and antifungal properties [26], there is little to no evidence to support these broader claims [26]. In fact, research states that vaginal boric acid suppositories should not be considered a preventive measure [26] and are only recommended as second-line treatment in specific cases of recurrent bacterial vaginosis or treatment-resistant yeast infections [27]. For example, in the United States, it is not a Food and Drug Administration–approved drug [28]. Despite this evidence, many women believe that boric acid is approved and is a natural, effective way to maintain vaginal health [28]. Negative cultural rhetoric that female genitalia are odorous, unattractive, unclean, or a cause for concern [29] may cause boric acid suppositories to be overused in an attempt to self-medicate or prevent perceived problems [28,30]. As there are a considerable number of vaginal boric acid suppository internet retailers [30], this is of particular concern for young women still developing an understanding of their bodies and body image [31], while navigating various influences from social media [32,33] and the new responsibility of making independent health decisions. Boric acid suppositories can cause abnormal discharge, burning, itching, inflammation, and heightened susceptibility to infection [25,29,34] or potentially delay appropriate medical care by masking other issues [30].

#### Fertility Testing

So-called “fertility” tests claiming to predict women’s fertility potential are increasingly being promoted by companies and influencers on social media. These tests can include measuring a range of different hormones, including biomarkers of ovarian reserve, such as the anti-Müllerian hormone or follicle-stimulating hormone. Many of these blood tests can now be ordered online and performed at home without a previous consultation with a physician. Although routinely used when undergoing fertility treatment to inform drug treatment protocols, these tests cannot predict a woman’s chance of conceiving, time to pregnancy, or reproductive timeline [35]. Despite lacking diagnostic value, online companies are increasingly selling these tests to women who have not even tried to become pregnant, capitalizing on their anxiety about their fertility [36,37]. Harms that can result from inappropriate testing include unnecessary anxiety about their ability to conceive, sometimes pressuring women to conceive before they are ready, or spending large amounts of money to freeze their eggs [38]. Conversely, it can also lead women to experience a false sense of security about delaying pregnancy.

#### Perimenopause, Menopause, and Postmenopause Products

##### Overview

Menopause—typically occurring between the ages of 45 and 55 years—is a normal event for women, characterized by the permanent end of the menstrual cycle caused by the cessation of reproductive hormone production in the ovaries. Before the final period, women undergo a phase of fluctuating ovarian function and hormone levels, known as perimenopause, which generally lasts for several years and may involve menstrual cycles with prolonged or heavy bleeding. Symptoms of menopause vary, but common symptoms include vasomotor symptoms (hot flushes and night sweats) and vaginal dryness. Growing awareness and broadening of acknowledged symptoms could represent positive strides for women’s empowerment and health [39]. However, this awareness has also given rise to a lucrative market for menopause-related products and treatments marketed directly to women on social media.

##### Perimenopause and Menopause Testing

Guidelines recommend that “Women over 45 years presenting with menopausal symptoms are diagnosed with perimenopause or menopause based on their symptoms alone, without confirmatory laboratory tests” [40], as there is no proven benefit to testing. However, biochemical testing and imaging are commonly promoted and undertaken—both through home testing kits and in clinical settings—to identify a woman’s menopausal status [41], despite guidelines recommending against such practices. Although such tests may be appropriate in women aged less than 45 years, for example, if premature menopause is suspected or other causes of oligomenorrhea or amenorrhea are excluded, they are considered unreliable, unnecessary, and costly for most women [42].

##### Menopause Treatments

Menopause treatments advertised on social media include supplements, dietary recommendations, estrogen gels, lifestyle coaching, and more conventional hormone therapies. Although conventional hormone therapies have proven effective in alleviating vasomotor symptoms, they are continuously advertised for alleviating other symptoms, such as weight gain, skin dryness, brain fog, and fatigue, for which robust evidence of their effect is lacking [43]. Hormone therapy is also marketed to women beyond what is recommended, potentially leading to unsafe use with serious side effects, such as increased risk of cardiovascular disease, cancer, and dementia [44]. For example, systemic hormone therapy (as opposed to topical therapy) is ineffective for the urinary symptoms of menopause and may in fact worsen the symptoms [45]. Furthermore, women are advertised topical progesterone creams and gels, despite no evidence to support their absorption through the skin [46]. Concerns have been raised about misleading claims regarding the safety and effectiveness of such products, as well as their potential to encourage women to bypass consultations with health care professionals when making treatment decisions [47,48].

##### Menopause Hormone Therapy for Disease Prevention

Women in their 60s are increasingly using social media [49], leading to increased exposure to fear-based messaging surrounding menopause and its long-term health impacts. Many influencers are promoting the use of hormone therapy not specifically to treat symptoms but for the prevention of dementia and cardiovascular disease, despite no reliable evidence [50] and no guideline recommendations to support the use of hormone therapy for this purpose [40,51]. Such treatment is associated with a long list of side effects and long-term harms, including risk of cancer and cardiovascular adverse events, as well as mood swings and irregular bleeding [52]. Thus, receiving hormone therapy to prevent future diseases is likely to have minimal health benefits and could be dangerous to women’s health.

### Data Collection

Consumer partners (1-2 across 5 age groups, ie, 20, 30, 40, 50, and 60 years relevant to each intervention target) based in Australia conducted keyword searches using their personal smartphones or tablets. To help account for the impact of the consumer’s previous search history and other social media accounts, consumer partners used new accounts matched to their gender and age, created for this study on 2 platforms (TikTok, Instagram, or Facebook). Keywords (Multimedia Appendix 1) related to the intervention were chosen based on the published literature for each health intervention and in consultation with consumers, and then refined through piloting on the social media platforms. The consumers searched using the keywords and then browsed the “For You”-style feed for the top posts, as displayed by the platform, until 100 eligible posts for each intervention on each platform were captured (200 posts total for each intervention). The sample size of the included posts is based on the concept of information power [53] and follows other health- and medical-related social media content analyses using manual searching and coding [22,23]. Posts were bookmarked (TikTok) and saved (Instagram and Facebook) in folders that were included and excluded on the platform. One researcher per intervention then screened the included and excluded posts for eligibility, and a second researcher checked if any disagreement arose between the consumer and the researcher. The links to the eligible posts were then extracted into Microsoft Excel.

Data collected about each social media post includes characteristics such as date posted, type of post (direct advertisement vs not), credentials of the account (eg, influencer or company with expertise health professional or company vs non–health professional), number of followers (ie, nano, micro, macro, midtier, or mega influencer), engagement cues (eg, views, likes, and comments), and target audience, if applicable.

In line with the study aims, we will also collect any information in the post that was related to the intervention’s benefits, harms, and caveats, use of relevant evidence, anecdotes, appeal strategies (eg, logos, pathos, and ethos), or narratives (eg, feminism, early detection, wellness, responsibility or moral imperatives), and financial interests of the posts account holders. Specific information relevant to each health intervention was also captured (eg, reference to specific evidence or claims made).

One to 2 weeks following the initial search and screening process, consumers logged into the account again and looked through the first 100 posts on the “For You” page on each platform. They then bookmarked (TikTok) or saved (Instagram and Facebook) any additional content related to the intervention in a separate folder [54]. The purpose of this step was to investigate whether the social media platform’s algorithm presented further content on the topics previously searched. As before, the captured posts were then discussed between the researchers to confirm inclusion and exclusion. The additional included posts will not be analyzed further.

### Data Analysis

All data from the post’s caption, on-screen text, and audio or video will be included in the analysis. The analysis will take both a deductive approach using a prespecified framework and an inductive approach, with independent extraction and review of the data undertaken by researchers and consumers to develop a list of recurring themes. The framework aligns with the study’s objectives of understanding how information about the above health interventions will be disseminated and promoted on social media by capturing post and influencer characteristics, references to specific health interventions, the use of scientific and medical claims, and persuasive techniques. The coding tool has been thoroughly tested for reliability and comprehensiveness and is informed by key domains and analysis of other coding tools used in previous media reporting and social media studies [22,23,55,56]. Two researchers will independently apply this coding tool to all the posts (in Microsoft Excel with preset responses, eg, yes, no, or unclear), with any discrepancies discussed and resolved through consensus, consulting a third researcher if necessary. On the basis of our experience with other similar social media content analyses [23], we will not calculate and report the interrater reliability (Cohen κ). As we expect the data to be highly imbalanced for the discussion of the benefits, harms, evidence, etc. (eg, discussion of benefits: 95% yes and 5% no or unclear), this will make the κ unstable and can underestimate true coder agreement [57].

Previous content analyses of social media have revealed novel perspectives and marketing strategies for health and medical tests [22,23]. This suggests the potential for a supplementary qualitative analysis of the posts to be included in this study. Following an initial familiarization phase to understand the dataset as a whole and its components as per the content analysis described above, the researchers will identify additional preliminary themes for each topic [58]. This will involve inductive qualitative coding of the posts, with preliminary themes identified organically; consumer perspectives will also be sought to help uncover new perspectives. The inductively identified preliminary themes will be applied to a subset of the data by several members of the team with iterative revision to refine the list. Once finalized, these themes will be added to our deductively defined, prespecified framework with the entire dataset double-coded, as described earlier. The coded data will be analyzed using a narrative approach, with frequencies for each category and theme calculated. Illustrative quotes will be included to contextualize the findings [59].

### Patient and Public Involvement

Eight consumer partners (1-2 per intervention) are involved in this research. In addition to providing valuable input into the health interventions selected for analysis, they have conducted the searches and will provide input into the analysis and interpretation of the findings. We aim to share a lay summary of the study findings with health consumer groups for dissemination in the wider community.

### Ethical Considerations

This study uses unequivocal public data. Data will be largely reported in aggregate form; however, in cases where specific excerpts (eg, quotes) are reported as examples, as is typical with the content analysis method [20], these will be short (eg, a few lines) and nonidentifiable (eg, no personal or professional information or reference will be given). Ethics approval for the study was obtained from The University of Sydney Human Research Ethics Committee, with a waiver of consent granted (2024/HE001041) because of the nonidentifiable nature of the data in any resulting publications.

## Results

### Progress to Date

Up to 1000 posts across all 5 interventions on TikTok, Instagram, and Facebook were searched and screened (from March to May 2025). One to two weeks after the initial search, an additional 500 posts (100 across each intervention) were screened for further content. Analysis is now underway.

### Dissemination

The findings will be disseminated via publications in peer-reviewed international medical journals and presentations at national and international conferences in 2025 and 2026. Deidentified datasets will be made available as per the publishing journals’ requirements or upon reasonable request to the study team.

## Discussion

### Anticipated Results

This study will provide novel insights into how information on various women’s health interventions and products, which currently lack robust evidence of benefit, are being disseminated and promoted on social media to women across their lifespan. Although it is acknowledged that the marketing of health interventions, including tests, treatments, and medical devices, currently exists on social media [23,60], specific data on how and by what means this are being disseminated to women across their lifespan are scarce.

This study has important strengths. The use of content analysis combines qualitative and quantitative methods to examine the content and context of social media posts, offering both structured and comprehensive insights into how 5 discrete and topical women’s health interventions are being marketed to women. This may then be extended to other non–evidence-based women’s health interventions. The analysis may also provide insights into how the algorithm feeds women this type of health information. In addition, consumers of various sociodemographic characteristics, including age, are involved in the study design, data collection, and interpretation of the study findings. Study limitations include the reliance on a small number of search terms to identify the study sample of posts about each intervention. The cross-sectional nature of the study means that we will only capture data at one time, and as is the case with all social media research, the timing of the search limits the reproducibility of the study findings. In addition, as noted in the Methods section, statistical tests of the interrater reliability (eg, κ) will not be calculated because of the expected instability of this measure in our study, which may affect the assessment of coding consistency across researchers. Instead, the reliability will be assessed manually, with any discrepancies discussed and resolved. Although there were no country restrictions on the included posts, they had to be presented in English. Furthermore, post comments and replies will also not be analyzed, which may miss additional information, including how potential misinformation is received, endorsed, or challenged. However, this is not the aim of our study and could be examined further in separate studies.

### Conclusions

Promotion of non-evidence-based health interventions to women on social media is a growing problem. Understanding the extent of the problem and the specific marketing strategies used is essential to develop approaches to mitigate potential harm and plan solutions. Findings from this study will provide information that may help women become aware of the issue and resist being unduly influenced or pressured by the marketing of low-value interventions. It will also provide the first comprehensive evidence on how non–evidence-based women’s health interventions are marketed across the lifespan using current examples on social media, which may help to inform health regulators of misleading marketing. Together, this will protect healthy women from becoming patients unnecessarily. This work will also help to minimize overuse, which takes health care resources from those in genuine need, threatening the sustainability of health systems.

## Appendix

Multimedia Appendix 1 List of keywords used to search each women’s health intervention.

## Data Availability

Data sharing is not applicable to this study as no datasets were generated or analyzed during this study.
