# Supplementary material for: Social Media Marketing of Non-Evidence-Based Women's Health Interventions: Protocol for a Content Analysis Using Participatory Research Methods
Source: JMIR Res Protoc. 2025 Oct 14;14:e76750. doi: 10.2196/76750 (PMC12569492; doi:10.2196/76750)
Supplement: Multimedia Appendix 1 [file resprot_v14i1e76750_app1.docx]

**Appendix. List of keywords used to search each women’s health intervention***

| **Int** | **Keyword 1** | **Keyword 2** | **Keyword 3** | **Keyword 4** |
| --- | --- | --- | --- | --- |
| **Boric acid suppositiories** | Instagram: Boricacid  TikTok: Boricacids | BVcure |  |  |
| **Fertility testing** | Fertility test | Egg count test |  |  |
| **Perimenopause and menopause testing** | Menopause testing | Perimenopause |  |  |
| **Menopause treatments** | Menopause treatment | Menopause lifestyle | Menopuase health | Menopuase HRT |
| **Menopause hormone therapy for disease prevention** | HRT prevention | HRT dementia | Hormone therapy prevention | Hormone therapy dementia |

*Platforms most common keywords based on piloting. As saturation in terms of eligible posts was reached (i.e., there were no eligible posts coming up) for keyword 1 and keyword 2 for Menopause treatments and menopause hormone therapy for disease prevention, two additional keywords were also included.
